# West Nile Virus Lineage 2 Vector Competence of Indigenous *Culex* and *Aedes* Mosquitoes from Germany at Temperate Climate Conditions

**DOI:** 10.3390/v12050561

**Published:** 2020-05-19

**Authors:** Cora M. Holicki, Ute Ziegler, Cristian Răileanu, Helge Kampen, Doreen Werner, Jana Schulz, Cornelia Silaghi, Martin H. Groschup, Ana Vasić

**Affiliations:** 1 Institute of Novel and Emerging Infectious Diseases, Friedrich-Loeffler-Institut, Federal Research Institute for Animal Health, 17493 Greifswald-Insel Riems, Germany; cora.holicki@fli.de (C.M.H.); ute.ziegler@fli.de (U.Z.); jana.schulz@fli.de (J.S.); martin.groschup@fli.de (M.H.G.); 2 Institute of Infectology, Friedrich-Loeffler-Institut, Federal Research Institute for Animal Health, 17493 Greifswald-Insel Riems, Germany; cristian.raileanu@fli.de (C.R.); helge.kampen@fli.de (H.K.); cornelia.silaghi@fli.de (C.S.); 3 Biodiversity of Aquatic and Semiaquatic Landscape Features, Leibniz-Centre for Agricultural Landscape Research, 15374 Muencheberg, Germany; doreen.werner@zalf.de

**Keywords:** West Nile virus, vector competence, transmission, arbovirus, *Culex pipiens*, *Aedes albopictus*

## Abstract

West Nile virus (WNV) is a widespread zoonotic arbovirus and a threat to public health in Germany since its first emergence in 2018. It has become of particular relevance in Germany in 2019 due to its rapid geographical spread and the detection of the first human clinical cases. The susceptibility of indigenous *Culex pipiens* (biotypes *pipiens* and *molestus*) for a German WNV lineage 2 strain was experimentally compared to that of Serbian *Cx. pipiens* biotype *molestus* and invasive German *Aedes albopictus*. All tested populations proved to be competent laboratory vectors of WNV. *Culex pipiens* biotype *pipiens* displayed the highest transmission efficiencies (40.0%–52.9%) at 25 °C. This biotype was also able to transmit WNV at 18 °C (transmission efficiencies of 4.4%–8.3%), proving that temperate climates in Central and Northern Europe may support WNV circulation. Furthermore, due to their feeding behaviors, *Cx. pipiens* biotype *molestus* and *Ae. albopictus* can act as “bridge vectors”, leading to human WNV infections.

## 1. Introduction

West Nile virus (WNV; *Flaviviridae*; *Flavivirus*) is the most dispersed zoonotic arbovirus worldwide and the causative agent of viral neurological diseases in susceptible animals and humans. The virus is maintained in an enzootic transmission cycle between ornithophilic mosquitoes as vectors and susceptible avian species as amplifying hosts [1]. Raptors (such as hawks and owls) and passeriform bird species (such as corvids) are highly susceptible, usually developing neuroinvasive diseases with high morbidity and mortality rates [2]. Rapid onset of disease and severe clinical symptoms are often associated with high viremia levels, sufficient to infect feeding mosquitoes and perpetuate the transmission cycle. However, amplifying hosts such as the American robin (*Turdus migratorius*) and the house sparrow (*Passer domesticus*) can show low mortality rates and still produce viremia levels high enough to infect mosquitoes [2,3]. Non-avian vertebrates, so-called “dead-end” hosts that are inadequate to pass on the virus to mosquitoes, can be infected with WNV via “bridge vectors” (mosquito species that feed on both birds and mammals) [1].

WNV was initially isolated in the West Nile district in Uganda in 1937 [4]. After extensive geographical dispersion mainly due to migratory birds [5], WNV is today endemic to every continent apart from Antarctica [1]. WNV lineage 1 was introduced into the United States in 1999 [6]. Through extensive infection of immunologically naïve avian hosts [7], it rapidly spread from the East to the West Coast within three years [1]. Unlike in the United States, WNV lineage 1 has been present in Europe and the Mediterranean at least since the late 1950s and was primarily associated with sporadic infections of low pathogenicity [1]. Only with the appearance of WNV lineage 2 in 2004 in several southern and southeastern European countries was there an increase in the severity and incidence of clinical cases in humans and equines [8,9,10]. In 2018, autochthonous WNV cases (12 in total) in resident wild and captive birds were recorded for the first time in Germany. The detected cases extended from the north to the south of eastern Germany (i.e., Munich to Rostock) [11,12]. In 2019, WNV incidence increased with numerous cases in birds and horses, including severe and fatal infections, primarily in Central and Eastern Germany [12]. In the same year, the first human infections were confirmed, with a total of five cases, which included WNV neuroinvasive disease [13].

Numerous studies indicated *Culex pipiens*, *Cx. quinquefasciatus*, and *Cx. tarsalis* to be principal vector species in the United States [14,15,16]. Similarly, *Cx. pipiens* plays a primary role in the transmission of WNV in Europe [17]. Vector competence studies with French [18], Italian [19], Spanish [20], Dutch [21,22,23,24,25], and German [26,27] *Cx. pipiens* populations proved their susceptibility to several WNV strains. Yet it remains unclear how efficient German mosquito populations are at transmitting WNV, especially regarding the two different *Cx. pipiens* biotypes (*pipiens* and *molestus*). These biotypes are known to differ in their seasonal activity and feeding-behavior (ornithophilic vs. mammalophilic) [28,29]. Furthermore, to better comprehend the spread of WNV in Germany and throughout Europe it is important to assess whether German mosquito species can transmit WNV under temperate climate conditions as found, for example, in Northern Europe. Finally, little is known about whether invasive mosquito species such as *Aedes albopictus* [30] play a role in WNV transmission in Central Europe.

The aim of this study was to investigate the vector competence of *Cx. pipiens* populations (biotypes *pipiens* and *molestus)* indigenous to Germany for the first German isolate of WNV lineage 2 [11] and to compare it to a Serbian (i.e., southern European) *Cx. pipiens* biotype *molestus* colony as well as an invasive German *Ae. albopictus* strain. Special focus was also placed on the influence of temperature on the vector competence of *Cx. pipiens* biotype *pipiens*.

## 2. Materials and Methods

### 2.1. Mosquito Origin and Rearing

Vector competence studies were performed with three mosquito taxa from Germany (*Cx. pipiens* biotypes *pipiens* and *molestus* and *Ae. albopictus*) and one colony (*Cx. pipiens* biotype *molestus*) from the Republic of Serbia (Table 1). *Culex pipiens* biotype *pipiens* egg rafts were collected in Brandenburg, Germany (“Schöneiche” and “Rehfelde” (S)), in 2018 and propagated in the laboratory, as well as in 2019 (“Groß Kreutz” (G)), where the F0 generation was directly used in the experiments. The *Cx. pipiens* biotype *molestus* colony was established in 2012 (“Wendland” (W), Lower Saxony, Germany) [26]. The *Ae. albopictus* colony was initiated from eggs acquired from an overwintering population in Jena (J), Thuringia, Germany, in 2016 [31]. For comparative purposes, a *Cx. pipiens* biotype *molestus* colony from Novi Sad (N), Republic of Serbia, established in 2012, was also tested.

For the identification of field-collected *Cx. pipiens* to species and biotype level, larvae hatching from each egg raft were tested by a real-time polymerase chain reaction (PCR) [32]. After pupation, the pupae were transferred into mosquito breeding cages (BugDorm; MegaView Science Co., Ltd., Taichung, Taiwan), and emerging adult mosquitoes were offered a 5% sugar solution ad libitum. They were kept at 24 °C ± 1 °C with a relative humidity of 60%–70% and a 16 h light/8 h dark photocycle. For the maintenance of established colonies, mosquitoes were artificially fed with chicken or bovine blood using the Hemotek Membrane Feeding System (Hemotek Ltd., Blackburn, United Kingdom). To assess whether mosquito populations were free from flaviviruses prior to the experiments, a minimum of two non-engorged females per population were examined via a WNV-specific reverse transcription quantitative real-time PCR (RT-qPCR) [33] and individual females were also tested in a SYBR^®^ Green-based quantitative real-time pan-flavivirus assay [34].

The WNV lineage 2 strain (GenBank accession no. MH924836) had been isolated from the brain of the first confirmed WNV infected bird, a great grey owl (*Strix nebulosa*), in Germany [11]. The virus was passaged three times alternately on Vero and *Ae. albopictus* C6/36 cell monolayers (the Collection of Cell Lines in Veterinary Medicine (CCLV), Friedrich-Loeffler-Institut (FLI), Greifswald-Insel Riems, Germany) and maintained in modified minimum essential medium (MEM) supplemented with 2% fetal calf serum (FCS). The last passage (P3) was performed on a Vero cell monolayer with a multiplicity of infection of 0.001. The virus stock was harvested four days post infection (dpi). The virus titer was quantified by means of an endpoint dilution assay on Vero cells and calculated with the Spearman–Karber algorithm [35]. The used stock had a titer of 9.3 log_10_ 50% tissue culture infective dose (TCID_50_) per mL. The virus stock was stored at −70 °C in 500 µL aliquots.

### 2.2. Mosquito Infection

Twenty-four hours prior to infection, female mosquitoes (5–14 days old) were sorted into transparent tubes and deprived of sugar solution and water. The infectious blood meal was composed of 90% heparinized chicken or bovine blood, based on the mosquito taxa’s feeding preference (Table 1), and 10% virus stock (i.e., 1:10 dilution of the virus stock with a titer of 9.3 log_10_ TCID_50_/mL). The blood was obtained from quarantine animals kept at the FLI, Greifswald-Insel Riems, Germany. As WNV-specific antibodies have not been detected in sentinel birds (mallards, *Anas platyrhynchos*) kept on the Isle of Riems (personal communication [36]), the chicken blood source was considered negative prior to the experiments. Bovine serum samples were tested to be free from WNV-specific antibodies using the ID Screen^®^ WN competition enzyme-linked immunosorbent assay (ELISA) (IDVet, Grabels, France). ATP (Merck, St. Louis, MO, USA) was added as a phagostimulant at a final concentration of 0.5 mM. In each tube, the mosquitoes were offered two cotton stick ends fully soaked in the infectious blood meal for three hours. The blood meal was titrated both before feeding and afterward from the cotton sticks on Vero cells to confirm the exact virus titer offered to the mosquitoes. Engorged females were transferred to new modified tubes under CO_2_-sedation. Two freshly engorged females per species were stored at −70 °C, to later confirm virus ingestion and construct a baseline for virus development in the mosquitoes via the WNV-specific RT-qPCR [33]. The remaining engorged mosquitoes were incubated under controlled conditions (18 °C, 25 °C, or 28 °C ± 1 °C, relative humidity of 80%–85%, 16 h light/8 h dark photocycle) in an incubator (MLR-352H-PE; Panasonic Corporation, Osaka, Japan) for 14/15 or 20/21 days, respectively. During this period, the mosquitoes were offered cotton pads soaked with 5% sugar solution ad libitum.

### 2.3. Mosquito Processing

After 14/15 or 20/21 days, respectively (i.e., salivation assay was performed on two consecutive days), mosquitoes were examined for virus presence in their bodies (thorax and abdomen), legs plus wings, and saliva. First, the mosquitoes were immobilized by detaching their legs and wings under CO_2_-anesthesia. Then, the forced salivation assay was performed according to Heitmann et al. [37], with saliva collection for 45–60 min. The mosquito bodies and legs plus wings were placed into separate 2 mL screw cap tubes with two 3-mm steel beads and 560 µL AVL viral lysis buffer and carrier RNA (QIAGEN, Hilden, Germany) and stored at −70 °C until RNA extraction. The saliva samples were inoculated directly onto a 96-well-plate cell monolayer of Vero cells. For virus inoculation, Vero cells were maintained in MEM supplemented with 2% FCS and 1% antimicrobials (gentamicin/amphotericin and penicillin/streptomycin; Merck, St. Louis, MO, USA). After seven days, the plates were fixed with 7.5% neutral buffered formalin (Carl Roth, Karlsruhe, Germany) and stained with crystal violet (Carl Roth, Karlsruhe, Germany). If a sample portrayed a distinct cytopathogenic effect, 140 µL of the cell culture supernatant was removed prior to staining and added to a 2-mL tube with 560 µL AVL viral lysis buffer and carrier RNA. RNA was extracted using the QIAamp Viral RNA Mini Kit (QIAGEN, Hilden, Germany), eluted in 50 µL of elution buffer, and stored at −70 °C. 

Mosquito bodies and legs plus wings were homogenized separately in the 2 mL screw cap tubes at 30 MHz for two minutes (TissueLyser II; QIAGEN, Hilden, Germany). Afterward, they were centrifuged in a 5430R centrifuge (Eppendorf, Hamburg, Germany) at room temperature for one minute at 13000 rpm. Nucleic acid was extracted from 200 µL of the supernatant with the BioSprint 96 (QIAGEN, Hilden, Germany) using the NucleoMag VET kit (MACHEREY-NAGEL, Düren, Germany). RNA extracts were eluted in 100 µL elution buffer and stored at −70 °C. Amplification of RNA (5 µL) from mosquito bodies, legs plus wings, and saliva cell culture supernatants was performed with a WNV-specific RT-qPCR assay [33] using the AgPath-ID One-Step RT-PCR Reagents (ThermoFischer Scientific, Darmstadt, Germany) and the CFX96TM Real-Time PCR Detection System (Bio-Rad Laboratories, Feldkirchen, Germany). For RNA quantification via RT-qPCR, a standard curve based on synthetic WNV RNA was run in parallel using 10-fold serial dilutions [33].

### 2.4. Vector Competence Indices

The feeding rate refers to the number of engorged females out of the total number of females exposed to the blood meal. The survival rate is the number of females surviving within a given period out of the total number of fed females. The infection rate describes the number of WNV-positive bodies in relation to the total number of mosquitoes examined. The dissemination rate is calculated as the number of specimens with WNV-positive legs plus wings out of the number of WNV-positive bodies. The transmission rate is the percentage of mosquitoes with infected bodies and legs plus wings that also had viable virus in their saliva. Transmission efficiency is the percentage of mosquitoes having viable virus in their saliva in relation to the total number of mosquitoes examined.

### 2.5. Data Analysis

All statistical analyses and graphical displays were performed with R version 3.6.0 (26 April 2019) [38] and the package “lsmeans” [39]. The effect of species, temperature, and/or sampling date (14/15 and 20/21 dpi), including their interactions (explanatory variables) on feeding, survival, infection, dissemination, and transmission rates and on transmission efficiencies (response variables), were investigated using generalized binomial regression models (glm). The final models were determined using stepwise backward elimination, leading to different models for the six response variables (Supplementary Materials Tables S1 and S2). Least-squares means [40] were used for testing linear contrasts among predictions with Tukey adjustment for *p*-values [41]. Results were deemed statistically relevant when the *p*-values (summarized in Supplementary Materials) were less than 0.05. Individual samples where the legs plus wings were virus-positive, but not the bodies, were not included in the analyses.

### 2.6. Ethics Statement

Blood for mosquito feeding was collected from animals kept at the FLI. Animals were held and sampled according to national and European legislation (Directive 2010/63/EU on the protection of animals used for scientific purposes). The procedures were approved by the competent authority of the Federal State of Mecklenburg-Western Pomerania, Germany (reference number: 7221.3-2-041/17, approved 12 Feb 2018).

## 3. Results

### 3.1. Feeding and Survival Rates

In total, 1217 mosquitoes were included in the WNV vector competence experiments (summarized in Table 2). Mosquito numbers were obtained via multiple replications of the infection experiments. The number of mosquitoes tested per time point fluctuates between mosquito populations, as the availability of mosquitoes for the saliva assays was strongly dependent on their feeding and survival rate. The 33.2% feeding rate of the field-acquired *Cx. pipiens* biotype *pipiens* (G) was significantly lower (*p* < 0.001) than the 82.8% rate of the laboratory-reared *Cx. pipiens* biotype *pipiens* (S). All other mosquito species had similar feeding rates varying between 53.1% and 56.7%. The *Ae. albopictus* population had the lowest survival rates 14/15 dpi (42.9%) and 14/15–20/21 dpi (58.1%). All other mosquito populations had survival rates ranging from 60.9% (*Cx. pipiens* biotype *molestus* from Serbia) to 90.4% (*Cx. pipiens* biotype *pipiens* from Germany) 14/15 dpi. Survival rates remained consistent 14/15–20/21 dpi, ranging from 65.6% (*Cx. pipiens* biotype *molestus* from Serbia) to 100.0% (*Cx. pipiens* biotype *pipiens* from Germany).

### 3.2. Vector Competence of Cx. pipiens and Ae. albopictus at 25 °C

In all infection experiments, the mosquitoes fed on a blood meal containing on average 7.3 (end of feeding period) to 8.2 (beginning of feeding period) log_10_ TCID_50_/mL of the German WNV strain. Infection, dissemination, and transmission rates and transmission efficiencies are summarized in Table 3. Both *Cx. pipiens* biotypes had high infection rates 14/15 dpi ranging from 64.5% to 100.0%. However, unlike *Cx. pipiens* biotype *pipiens* (S and G), the infection rates of *Cx. pipiens* biotype *molestus* (W and N) decreased to 6.7% and 15.0% 20/21 dpi. This resulted in statistically significant differences between the infection rates of both *Cx. pipiens* biotype *pipiens* colonies (S and G) and those of the German *Cx. pipiens* biotype *molestus* (W) (*p* = 8.1 × 10^−3^ and *p* = 2.5 × 10^−2^, respectively) and Serbian *Cx. pipiens* biotype *molestus* (N) (*p* = 1.5 × 10^−2^ and *p* = 3.9 × 10^−3^, respectively) 20/21 dpi. *Aedes albopictus* was the only species that had low infection rates (9.8% and 20.0%) at both sampling dates (14/15 and 20/21 dpi). These were significantly lower than those of the two *Cx. pipiens* biotype *pipiens* colonies (S and G) (*p* = 1.1 × 10^−2^ and *p* = 5.8 × 10^−3^, respectively) 20/21 dpi. However, the dissemination and transmission rates did not differ significantly between the different mosquito populations. All mosquito populations derived from field-collected egg rafts were tested negative for WNV prior to the infection experiments.

**Figure 1:**
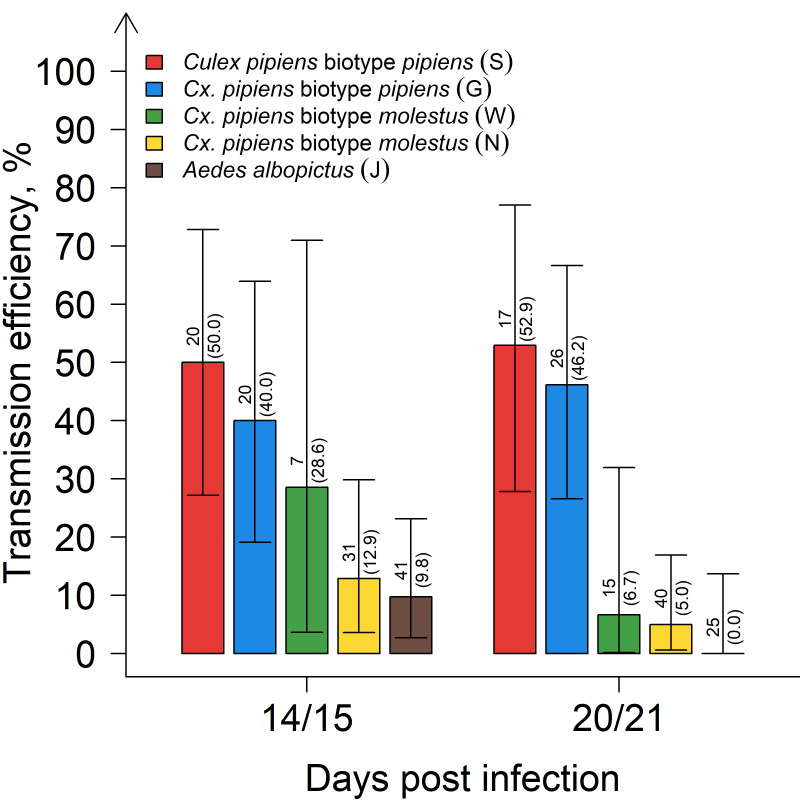
Transmission efficiencies for *Cx. pipiens* biotype *pipiens*, *Cx. pipiens* biotype *molestus*, and *Ae. albopictus* 14/15 and 20/21 days post infection at an incubation temperature of 25 °C. Error bars represent 95% confidence intervals. The number above each bar indicates the number of mosquitoes tested per species/time point, and in brackets, the percentage tested WNV-positive.

When comparing the transmission efficiencies (Figure 1) of the German *Cx. pipiens* biotype *pipiens* (S and G) with the Serbian *Cx. pipiens* biotype *molestus* (N), significant differences were observed independent of the sampling date (*p* < 0.001, respectively). Differences were also found between the German *Cx. pipiens* biotype *pipiens* (S and G) and the German *Cx. pipiens* biotype *molestus* (W), even though these were not statistically significant (*p* = 5.4 × 10^−2^ and *p* = 1.5 × 10^−1^, respectively). Transmission efficiencies of *Ae. albopictus* were also significantly lower than those of the *Cx. pipiens* biotype *pipiens* (S and G) (*p* < 0.001, respectively).

**Figure 2:**
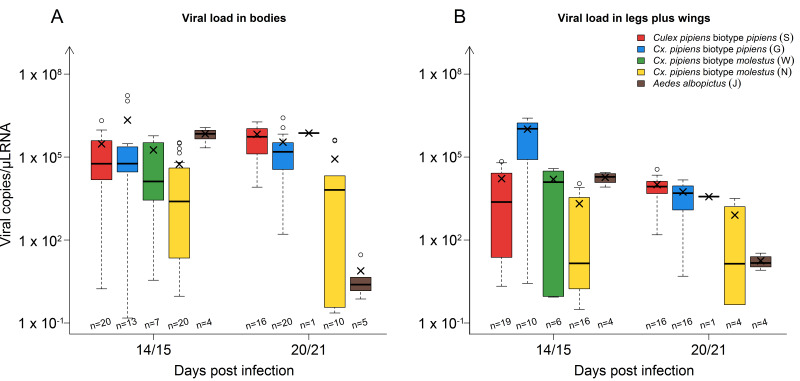
Viral load in mosquito bodies (**A**) and legs plus wings (**B**) 14/15 and 20/21 days post infection in the different strains: *Culex pipiens* biotype *pipiens*, *Cx. pipiens* biotype *molestus*, and *Aedes albopictus*. The horizontal black lines represent the medians, the black ×’s the means, the boxes show the interquartile ranges, and the whiskers the minimum and maximum values. Data falling outside the interquartile ranges are plotted as outliers (o). The number of samples tested per species/time point are indicated above the x-axis.

The viral loads (virus RNA copies/µL RNA) in the mosquito bodies and legs plus wings 14/15 and 20/21 dpi are depicted in Figure 2. The mean viral load in the *Culex* strains remained fairly stable from 14/15 to 20/21 dpi. The values in the bodies ranged from 5.5 × 10^4^ to 2.2 × 10^6^ copies/µL RNA and in the legs plus wings from 8.0 × 10^2^ to 1.0 × 10^6^ copies/µL RNA. *Aedes albopictus*, however, showed a time-dependent decline in the mean viral load both in the mosquito bodies (6.9 × 10^5^ to 7.7 copies/µL RNA) and in the legs plus wings (1.9 × 10^4^ to 1.8 × 10^1^ copies/µL RNA). The viral loads found in the mosquitoes directly after blood feeding ranged between 1.8 × 10^1^ to 8.8 × 10^2^ (cycle threshold of 24.4–30.1). By contrast, all non-engorged day 0 samples lacked flavivirus-specific RNA.

### 3.3. Vector Competence of Cx. pipiens Biotype pipiens at Three Different Temperature Regimes (18 °C, 25 °C, and 28 °C)

**Figure 3:**
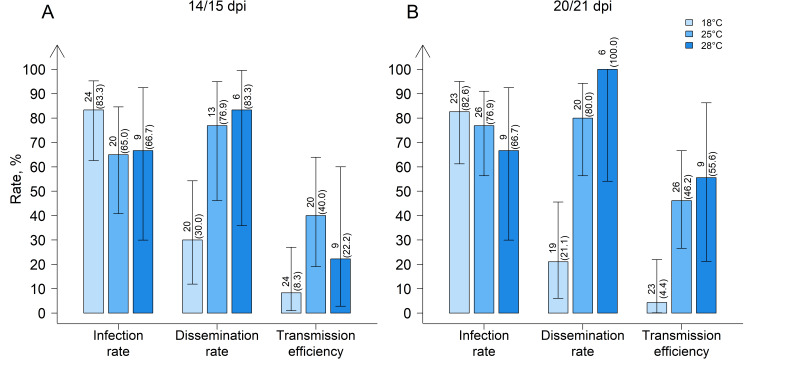
Infection rate, dissemination rate, and transmission efficiency of field-collected *Culex pipiens* biotype *pipiens* from “Groß Kreutz”, Brandenburg, Germany, (**A**) 14/15 and (**B**) 20/21 days post infection (dpi) at three different temperature regimes. Error bars represent 95% confidence intervals. The number above each bar indicates the number of mosquitoes tested per species/time point, and in brackets, the percentage tested WNV-positive.

German *Cx. pipiens* biotype *pipiens* from “Groß Kreutz” (G), Brandenburg, showed higher dissemination and transmission efficiencies at 25 °C and 28 °C than at 18 °C (Figure 3). The dissemination rates (14/15 and 20/21 dpi) at 28 °C (83.3% and 100.0%) and 25 °C (76.9% and 80.0%) were significantly higher than at 18 °C (30.0% and 21.1%) (*p* < 0.001 and *p* = 5.0 × 10^−3^, respectively). Similarly, the transmission efficiencies (14/15 and 20/21 dpi) were significantly higher at 28 °C (22.2% and 55.6%) and 25 °C (40.0% and 46.2%) than at 18 °C (8.3% and 4.4%) (*p* < 0.001 and *p* = 1.0 × 10^−2^, respectively).

## 4. Discussion

This study demonstrates that German *Cx. pipiens* mosquitoes and Serbian *Cx. pipiens* biotype *molestus* fulfill the criteria for WNV vectors, portraying high virus susceptibility and efficient virus transmission, even at temperate climate conditions (e.g., 18 °C), which are common in Germany during the summer season [42]. The results also revealed, for the first time, the WNV vector competence of an established *Ae. albopictus* population in Germany [31]. The virus titers in the blood meals used in this study of 7.3–8.2 log_10_ TCID_50_/mL are commonly found in viremic birds in nature, with most species, however, not reaching peak viremia levels above 8 log_10_ TCID_50_/mL [17]. Therefore, the results of this study can relate to typical scenarios found in the field. When discussing these results, one must, however, bear in mind that the different blood sources used (chicken vs. bovine) may have had a modest effect on the vector competence of mosquito populations. The same is true for the laboratory colonization of the mosquito colonies used in this study [17].

*Culex pipiens* biotype *pipiens* is highly ubiquitous throughout Germany and, due to its ornithophilic feeding preference, suitable as a primary amplifier in the enzootic transmission cycle of WNV [32]. In general, the high susceptibility of *Cx. pipiens* biotype *pipiens* for WNV described within this study correlates with the results from several other studies of European mosquitoes [17]. These studies have described a virus and/or mosquito strain-dependent difference in the vector competence of mosquitoes [17]. This current study also reinforces this idea as the transmission efficiencies of the two German *Cx. pipiens* biotype *pipiens* populations (50.0% and 40.0% 14/15 dpi, respectively) infected with the German WNV lineage 2 strain were higher than those of Dutch (10.3% [25], 6% [17,23], and 10% [24]) and Italian (2% [24]) populations of the same species infected with a Greek WNV lineage 2 strain (GenBank accession no. HQ537483) after incubation at 23 °C or 25 °C. The transmission efficiencies of German *Cx. pipiens* biotype *pipiens* after infection with an Italian WNV lineage 1 strain (GenBank accession no. HM991273/HM641225) were even lower [27]. Further evidence supporting the role of *Culex pipiens* biotype *pipiens* as a WNV vector in the field was provided in 2019, where WNV was detected for the first time in German mosquitoes (in five *Cx. pipiens* biotype *pipiens* pools and two *Cx. pipiens* biotype *pipiens*/*molestus* pools) [43]. Keeping all these variations in mind, *Cx. pipiens* can be designated as an important vector for WNV in Europe.

The German and Serbian *Cx. pipiens* biotype *molestus* on the other hand had lower transmission efficiencies for WNV than the two *Cx. pipiens* biotype *pipiens* colonies, particularly at a later point in time post infection. Interestingly though, the tested *Cx. pipiens* biotype *molestus* populations showed infection rates (100.0% and 64.5%, respectively) and transmission efficiencies (28.6% and 12.9%, respectively) that were higher than those of Dutch *Cx. pipiens* biotype *molestus* infected with a Greek WNV lineage 2 strain (GenBank accession no. HQ537483.1) (24% and 10%, respectively) 14/15 dpi [17,23]. Although *Cx. pipiens* biotype *molestus* does not appear to be an essential amplifier in the transmission cycle between birds, it may act as a “bridge vector” due to its mammalophilic feeding-preference, infecting humans with WNV. Nonetheless, the limited number of females tested enables only a preliminary indication of the vector competence of this biotype.

*Aedes albopictus* is a vastly spreading, invasive mosquito species from tropical Asia and the Pacific and is a known secondary vector of many arboviruses [44,45,46,47]. In this study, the *Ae. albopictus* population from Jena, Germany, was susceptible to WNV lineage 2, although with significantly lower infection rates and transmission efficiencies (9.8% 14/15 dpi for both) than *Cx. pipiens* biotype *pipiens*. It was also less susceptible to the German WNV lineage 2 strain than Italian *Ae. albopictus* to an Italian WNV lineage 1 strain (GenBank accession no. HQ537483.1 [48]), with infection rates of 9.8% compared to 80% [45]. The difference, however, may have been due to a lower incubation temperature (25 °C vs. 27 °C). Furthermore, the Italian *Ae. albopictus* infected with the Italian WNV lineage 1 strain could still transmit virus 21 dpi [45] unlike the *Ae. albopictus* in this study. The German *Ae. albopictus* showed a strong decline in viral RNA copies/µL in the bodies and legs plus wings from 14/15 to 20/21 dpi. This could indicate a decline of intracellular virus replication in the mosquitoes followed by an absence of infectious virus particles in their saliva. Even though *Ae. albopictus* showed the lowest transmission efficiencies, the species may still be a relevant vector of WNV for humans due to its high abundance in human settlements and the intake of multiple blood meals from different hosts in a short period of time [49]. *Aedes albopictus* should therefore be considered a WNV vector in regions of Germany, where it succeeded in establishing itself and has reached high seasonal population densities [50].

With 19.3 °C and 19.2 °C, respectively, the average summer temperatures in 2018 and 2019 were unusually high for Germany, i.e., 2.1–2.2 °C warmer than normal (1981–2010) [42]. For a total of 74 and 52 days, respectively, the maximum daily temperature reached or exceeded 25 °C [51]. To better understand the ongoing epidemiology and predict the spread of WNV throughout Germany, it is essential to further test the temperature dependency of WNV transmission per mosquito species and population. This study focused on the most susceptible taxon, *Cx. pipiens* biotype *pipiens*. Transmission efficiencies were similar at incubation temperatures of 25 °C and 28 °C, however, they were significantly lower at 18 °C. This could primarily be due to the positive correlation between intracellular virus replication and temperature [17,52]. It is also possible that the midgut and salivary gland escape barriers only impede WNV vector competence at low temperatures but not at 25 °C or 28 °C. High temperatures can destabilize the midgut barrier and induce changes in the regulation of a mosquito’s immune system and RNA-interference pathways [53]. Other vector competence studies have described a complete lack of WNV transmission at incubation temperatures below 21 °C [27]. This was refuted by our results where several mosquitoes had WNV in their saliva after incubation at 18 °C. More frequent climate extremes associated with increased temperatures are anticipated in the near future [54] and will probably result in WNV propagation throughout Germany due to longer WNV transmission and mosquito breeding seasons with greater population densities [52].

## 5. Conclusions

Both *Cx. pipiens* biotypes (*pipiens* and *molestus*) and *Ae. albopictus* were proven vector-competent for the German WNV lineage 2 strain in the laboratory, suggesting a role as WNV vectors also in the field. *Culex pipiens* biotype *pipiens* appears to be highly adapted to temperate climate conditions found in Central and Northern Europe, still supporting WNV transmission at 18 °C. The expansion of WNV throughout Germany is facilitated through the expected increasing temperatures and the presence of highly potent WNV vectors and susceptible hosts in Germany.

## Figures and Tables

**Table 1:** Mosquito taxa and blood sources used in the infection experiments.

Mosquito Taxon	Collection Site	Year of Collection	Laboratory Colony	Field-Collected	Blood Source
*Culex pipiens* biotype *pipiens*	“Schöneiche“ and “Rehfelde” (S), Brandenburg, Germany	2018	X		Chicken
	“Groß Kreutz” (G), Brandenburg, Germany	2019		X	Chicken
*Cx. pipiens* biotype *molestus*	“Wendland” (W), Lower Saxony, Germany	2012	X		Bovine
	Novi Sad (N), Republic of Serbia	2012	X		Bovine
*Aedes albopictus*	Jena (J), Thuringia, Germany	2016	X		Bovine

**Table 2:** Mosquito feeding and survival rates, up to 14/15 days post infection (dpi) and 14/15–20/21 dpi, with German West Nile virus lineage 2 at different temperatures. CI stands for confidence interval.

Mosquito Species	Incubation Temperature (°C)	Feeding Rate (%) (95% CI)	Survival Rate 14/15 dpi (Excluding Day 0 Samples) (%) (95% CI)	Survival Rate 14/15–20/21 dpi (Excluding Day 0 Samples and Mosquitoes Tested 14/15 dpi) (%) (95% CI)
*Culex pipiens* biotype *pipiens* (S)	25	48/58 (82.8) (70.6–91.4)	41/46 (89.1) (76.4–96.4)	17/21 (81.0) (58.1–94.6)
*Cx. pipiens* biotype *pipiens* (G)	18	147/443 (33.2) (28.8–37.8)	47/52 (90.4) (77.8–95.9)	23/23 (100.0) (72.6–97.8)
	25		49/55 (89.1) (79.0–96.8)	26/29 (89.7) (85.2–100.0)
	28		21/30 (70.0) (50.6–85.3)	9/12 (75.0) (42.8–94.5)
*Cx. pipiens* biotype *molestus* (W)	25	34/64 (53.1) (40.2–65.7)	23/30 (76.7) (57.7–90.1)	15/16 (93.8) (69.8–99.8)
*Cx. pipiens* biotype *molestus* (N)	25	159/299 (53.2) (47.3–58.9)	92/151 (60.9) (52.7–68.8)	40/61 (65.6) (52.3–77.3)
*Aedes albopictus* (J)	25	200/353 (56.7) (51.3–61.9)	84/196 (42.9) (35.8–50.1)	25/43 (58.1) (42.1–73.0)

**Table 3:** Infection, dissemination, and transmission rates and transmission efficiencies 14/15 and 20/21 days post infection at an incubation temperature of 25 °C. CI stands for confidence interval.

Mosquito Species	Days Post Infection	Infection Rate (%) (95% CI)	Dissemination Rate (%) (95% CI)	Transmission Rate (%) (95% CI)	Transmission Efficiency (%) (95% CI)
*Culex pipiens* biotype *pipiens* (S)	14/15	20/20 (100.0) (83.2–100.0)	19/20 (95.0) (75.1–99.9)	10/19 (52.6) (28.9–75.6)	10/20 (50.0) (27.2–72.8)
	20/21	16/17 (94.1) (71.3–99.9)	16/16 (100.0) (79.4–100.0)	9/16 (60.0) (29.9–80.2)	9/17 (52.9) (27.8–77.0)
*Cx. pipiens* biotype *pipiens* (G)	14/15	13/20 (65.0) (40.8–84.6)	10/13 (76.9) (46.2–95.0)	8/10 (80.0) (44.4–97.5)	8/20 (40.0) (19.1–63.9)
	20/21	20/26 (76.9) (56.4–91.0)	16/20 (80.0) (56.3–94.3)	12/16 (75.0) (47.6–92.7)	12/26 (46.2) (26.6–66.6)
*Cx. pipiens* biotype *molestus* (W)	14/15	7/7 (100.0) (59.0–100.0)	6/7 (85.7) (42.1–99.6)	2/6 (33.3) (4.3–77.7)	2/7 (28.6) (3.7–71.0)
	20/21	1/15 (6.7) (0.2–31.9)	1/1 (100.0) (2.5–100.0)	1/1 (100.0) (2.5–100.0)	1/15 (6.7) (0.2–31.9)
*Cx. pipiens* biotype *molestus* (N)	14/15	20/31 (64.5) (45.4–80.8)	16/20 (80.0) (56.3–94.3)	4/16 (25.0) (7.3–52.4)	4/31 (12.9) (3.6–29.8)
	20/21	10/40 (15.0) (12.7–41.2)	4/10 (40.0) (12.2–73.8)	2/4 (50.0) (6.8–93.2)	2/40 (5.0) (0.6–16.9)
*Aedes albopictus* (J)	14/15	4/41 (9.8) (2.7–23.1)	4/4 (100.0) (39.8–100)	4/4 (100.0) (39.8–100)	4/41 (9.8) (2.7–23.1)
	20/21	5/25 (20.0) (6.8–40.7)	4/5 (80.0) (28.4–99.5)	0/4 (0.0) (0.0–60.2)	0/25 (0.0) (0.0–13.7)

## Supplementary Materials

The following are available online at https://www.mdpi.com/1999-4915/12/5/561/s1, Table S1: Overview on explanatory variables included in the final generalized binomial regression models (glm) for the investigated rates at 25 °C, Table S2: Overview on explanatory variables included in the final generalized binomial regression models (glm) for *Culex pipiens* biotype *pipiens* (G) at three different temperature regimes for the investigated rates, Table S3: *p*-values of the fixed effects in the least-square means analysis when comparing the rates between all species at 25 °C in the final generalized binomial regression models specified in Supplementary Materials Table S1. *p*-value adjustment was performed using the Tukey method, Table S4: *p*-values of the interaction term in the least-square means analysis for the infection and transmission rate at 25 °C, Table S5: *p*-values of the fixed effect temperature in the least-square means analysis when comparing the rates at temperatures of 18 °C, 25 °C, and 28 °C in the final generalized binomial regression models specified in Supplemental Table S2. *p*-value adjustment was performed using the Tukey method.
