# Supplementary material for: West Nile Virus Lineage 2 Vector Competence of Indigenous Culex and Aedes Mosquitoes from Germany at Temperate Climate Conditions
Source: Viruses. 2020 May 19;12(5):561. doi: 10.3390/v12050561 (PMC7291008; doi:10.3390/v12050561)
Supplement: Supplementary file 1 [file viruses-12-00561-s001.pdf]

Supplementary Materials

Table S1. Overview on explanatory variables included in the final generalized binomial regression models (glm) for the investigated rates at 25 °C.

	Response variables					
**Explanatory variables**	**Feeding rate**	**Survival rate**	**Infection rate**	**Dissemination rate**	**Transmission rate**	**Transmission efficiency**
**Species**	x	x	x	x	x	x
**Days post infection**	/	x	x	x	x	/
**Interaction term: Species: days post infection**	/	/	x	/	x	/

Table S2. Overview on explanatory variables included in the final generalized binomial regression models (glm) for *Culex pipiens* biotype *pipiens* (G) at three different temperature regimes for the investigated rates.

	Response variables			
**Explanatory variables**	**Infection rate**	**Dissemination rate**	**Transmission rate**	**Transmission efficiency**
**Temperature**	x	x	x	x
**Days post infection**	/	/	/	/
**Interaction term: Temperature: days post infection**	/	/	/	/

Table S3. *p*-values of the fixed effects in the least-square means analysis when comparing the rates between all species at 25 °C in the final generalized binomial regression models specified in Supplementary Materials Table S1. *p*-value adjustment was performed using the Tukey method.

**Fixed effects:**							
		**Feeding rate**	**Survival rate**	**Infection rate⁺**	**Dissemination rate**	**Transmission rate⁺**	**Transmission efficiency**
	**contrast**	***p*-value**	***p*-value**	***p*-value**	***p*-value**	***p*-value**	***p*-value**
**Species**	AA_J - CPM_N	9.01×10^{-1}	7.88×10^{-3}*	4.66×10^{-3}*	6.50×10^{-1}	1.00	9.84×10^{-1}
	AA_J - CPM_W	9.85×10^{-1}	4.05×10^{-4}*	1.00	9.98×10^{-1}	1.00	8.02×10^{-1}
	AA_J - CPP_G	5.12×10^{-10}*	2.39×10^{-8}*	3.09×10^{-7}*	9.67×10^{-1}	1.00	3.04×10^{-4}*
	AA_J - CPP_S	3.22×10^{-3}*	1.57×10^{-6}*	1.00	8.74×10^{-1}	1.00	4.83×10^{-5}*
	CPM_N - CPM_W	1.00	8.79×10^{-2}	1.00	8.69×10^{-1}	1.00	9.54×10^{-1}
	CPM_N - CPP_G	7.29×10^{-7}*	2.36×10^{-4}*	4.62×10^{-2}*	6.57×10^{-1}	1.24×10^{-1}	4.39×10^{-4}*
	CPM_N - CPP_S	7.97×10^{-4}*	3.90×10^{-3}*	1.00	5.14×10^{-2}	8.14×10^{-1}	5.98×10^{-5}*
	CPM_W - CPP_G	1.91×10^{-2}*	8.20×10^{-1}	1.00	9.99×10^{-1}	1.00	1.45×10^{-1}
	CPM_W - CPP_S	6.77×10^{-3}*	9.75×10^{-1}	1.00	7.23×10^{-1}	1.00	5.35×10^{-2}
	CPP_G - CPP_S	3.67×10^{-9}*	9.88×10^{-1}	1.00	2.90×10^{-1}	3.83×10^{-1}	9.53×10^{-1}
**Days post infection**	14/15 – 20/21	NA	9.80×10^{-2}	9.90×10^{-1}	2.13×10^{-1}	9.97×10^{-1}	NA

*$p < 0.05$. ⁺Results for fixed effects may be misleading due to involvement in interactions (Supplementary Materials Table S4). CPP_G: *Culex pipiens* biotype *pipiens* from "Groß Kreutz", Brandenburg, Germany. CPP_S: *Cx. pipiens* biotype *pipiens* from "Schöneiche" and "Rehfelde", Brandenburg, Germany. CPM_W: *Cx. pipiens* bioytpe *molestus* from "Wendland", Lower Saxony, Germany. CPM_N: *Cx. pipiens* biotype *molestus* from Novi Sad, Republic of Serbia. AA_J: *Aedes albopictus* from Jena, Thuringia, Germany.

Table S4. *p*-values of the interaction term in the least-square means analysis for the infection and transmission rate at 25 °C.

**Interactions:**			
		**Infection rate**	**Transmission rate**
**Species:days post infection**	**contrast**	***p*-value**	***p*-value**
	AA_J,14/15 - CPM_N,14/15	5.35×10^{-4}*	1.00
	AA_J,14/15 - CPM_W,14/15	1.00	1.00
	AA_J,14/15 - CPP_G,14/15	2.22×10^{-3}*	1.00
	AA_J,14/15 - CPP_S,14/15	1.00	1.00
	AA_J,14/15 - AA_J,20/21	9.79×10^{-1}	1.00
	AA_J,14/15 - CPM_N,20/21	7.62×10^{-1}	1.00
	AA_J,14/15 - CPM_W,20/21	1.00	1.00
	AA_J,14/15 - CPP_G,20/21	4.66×10^{-5}*	1.00
	AA_J,14/15 - CPP_S,20/21	6.63×10^{-4}*	1.00
	CPM_N,14/15 - CPM_W,14/15	1.00	1.00

CPM_N,14/15 - CPP_G,14/15	1.00	2.48×10^{-1}
CPM_N,14/15 - CPP_S,14/15	1.00	8.33×10^{-1}
CPM_N,14/15 - AA_J,20/21	$4.85 \times 10^{-2*}$	1.00
CPM_N,14/15 - CPM_N,20/21	$3.96 \times 10^{-2*}$	9.95×10^{-1}
CPM_N,14/15 - CPM_W,20/21	9.48×10^{-2}	1.00
CPM_N,14/15 - CPP_G,20/21	9.92×10^{-1}	1.77×10^{-1}
CPM_N,14/15 - CPP_S,20/21	6.12×10^{-1}	7.60×10^{-1}
CPM_W,14/15 - CPP_G,14/15	1.00	7.52×10^{-1}
CPM_W,14/15 - CPP_S,14/15	1.00	9.98×10^{-1}
CPM_W,14/15 - AA_J,20/21	1.00	1.00
CPM_W,14/15 - CPM_N,20/21	1.00	1.00
CPM_W,14/15 - CPM_W,20/21	1.00	1.00
CPM_W,14/15 - CPP_G,20/21	1.00	7.84×10^{-1}
CPM_W,14/15 - CPP_S,20/21	1.00	9.95×10^{-1}
CPP_G,14/15 - CPP_S,14/15	1.00	9.27×10^{-1}
CPP_G,14/15 - AA_J,20/21	9.84×10^{-2}	1.00
CPP_G,14/15 - CPM_N,20/21	1.08×10^{-1}	9.86×10^{-1}
CPP_G,14/15 - CPM_W,20/21	1.15×10^{-1}	1.00
CPP_G,14/15 - CPP_G,20/21	9.97×10^{-1}	1.00
CPP_G,14/15 - CPP_S,20/21	6.68×10^{-1}	9.71×10^{-1}
CPP_S,14/15 - AA_J,20/21	1.00	1.00
CPP_S,14/15 - CPM_N,20/21	1.00	1.00
CPP_S,14/15 - CPM_W,20/21	1.00	1.00
CPP_S,14/15 - CPP_G,20/21	1.00	9.43×10^{-1}
CPP_S,14/15 - CPP_S,20/21	1.00	1.00
AA_J,20/21 - CPM_N,20/21	1.00	1.00
AA_J,20/21 - CPM_W,20/21	9.86×10^{-1}	1.00
AA_J,20/21 - CPP_G,20/21	$5.81 \times 10^{-3*}$	1.00
AA_J,20/21 - CPP_S,20/21	$1.06 \times 10^{-2*}$	1.00
CPM_N,20/21 - CPM_W,20/21	9.27×10^{-1}	1.00
CPM_N,20/21 - CPP_G,20/21	$3.94 \times 10^{-3*}$	9.95×10^{-1}
CPM_N,20/21 - CPP_S,20/21	$1.46 \times 10^{-2*}$	1.00
CPM_W,20/21 - CPP_G,20/21	$2.47 \times 10^{-2*}$	1.00
CPM_W,20/21 - CPP_S,20/21	$8.07 \times 10^{-3*}$	1.00
CPP_G,20/21 - CPP_S,20/21	9.32×10^{-1}	9.84×10^{-1}

*$p < 0.05$. CPP_G: *Culex pipiens* biotype *pipiens* from "Groß Kreutz", Brandenburg, Germany. CPP_S: *Cx. pipiens* biotype *pipiens* from "Schöneiche" and "Rehfelde" Brandenburg, Germany. CPM_W: *Cx. pipiens* bioytpe *molestus* from "Wendland" Lower Saxony, Germany. CPM_N: *Cx. pipiens* biotype *molestus* from "Novi Sad", Republic of Serbia. AA_J: *Aedes albopictus* from Jena, Germany.

Table S5. *p*-values of the fixed effect temperature in the least-square means analysis when comparing the rates at temperatures of 18 °C, 25 °C, and 28 °C in the final generalized binomial regression models specified in Supplemental Table 2. *p*-value adjustment was performed using the Tukey method.

**Fixed effect:**						
		**Survival rate**	**Infection rate**	**Dissemination rate**	**Transmission rate**	**Transmission efficiency**
	**contrast**	***p*-value**	***p*-value**	***p*-value**	***p*-value**	***p*-value**
**Temperature (°C)**	18 - 25	6.46×10^{-1}	4.04×10^{-1}	6.95×10^{-5}*	3.66×10^{-2}*	8.14×10^{-4}*
	18 - 28	7.74×10^{-3}*	3.37×10^{-1}	5.01×10^{-3}*	2.87×10^{-1}	1.02×10^{-2}*
	25 - 28	3.78×10^{-2}*	9.16×10^{-1}	6.01×10^{-1}	6.87×10^{-1}	9.40×10^{-1}

*$p < 0.05$.
